# A WAY OF COMMUNICATING...WHICH DOESN’T MAKE THEM FEEL THREATENED: CANCER SURVIVORSHIP IN AGING BLACK/LATINX PEOPLE

**DOI:** 10.1093/geroni/igad104.3156

**Published:** 2023-12-21

**Authors:** Chizobam Nweke, Candidus “Candi” Nwakasi

**Affiliations:** University of Connecticut, Storrs, Connecticut, United States; University of Connecticut, Storrs, Connecticut, United States

## Abstract

Background. About 2 million people are diagnosed with cancer each year in the US and minoritized groups such as Black and Latinx people share a significant burden of the disease. To better understand how to improve the quality of life of an increasingly diverse population of aging cancer survivors, this study explored the cancer survivorship experiences of older adults in Black and Latinx people in the U.S. Method. A qualitative descriptive approach was used on a sample of 17 participants (Mage 64 years), 6 Black and 12 Latinx cancer survivors, at various stages of cancer treatment. Open-ended, semi-structured interviews were administered to the participants. We used reflexive thematic analysis for data analysis. Results. The following themes were identified from the analysis: cancer is (not) stigmatizing, effects of race/ethnicity on survivorship, and being an immigrant and survivorship. These themes enable a better perception of enablers and barriers to resilience among Black and Latinx cancer survivors. Conclusion. The findings of this study will increase the knowledge on sociocultural factors that influence cancer survivorship experiences in the US. The implications on resilience and access to cancer care will be discussed.

